# Spatial and temporal modeling of the global burden of Cutaneous Leishmaniasis in Brazil: A 21-year ecological study

**DOI:** 10.1371/journal.pntd.0012668

**Published:** 2024-11-20

**Authors:** Erica Santos dos Reis, Wandklebson Silva Paz, Rosália Elen Santos Ramos, Caíque Jordan Nunes Ribeiro, Laiza Santos Biano, Márcio Bezerra-Santos, Camila Indiani de Oliveira, Michael Wheeler Lipscomb, Tatiana Rodrigues de Moura

**Affiliations:** 1 Health Sciences Graduate Program, Federal University of Sergipe, Palestina, Aracaju, Sergipe, Brazil; 2 Medicine Tropical Graduate Program, Federal University of Pernambuco, Recife, Pernambuco, Brazil; 3 Department of Nursing, Federal University of Sergipe, Lagarto, Sergipe, Brazil; 4 Graduate Program in Nursing, Federal University of Sergipe, São Cristóvão, SE, Brazil; 5 Department of Physiology, Postgraduate Program in Physiological Sciences, Federal University of Sergipe, São Cristóvão, SE, Brazil; 6 Medical Sciences and Nursing Complex, Federal University of Alagoas, Arapiraca, Alagoas, Brazil; 7 Post-graduate Programme in Health Sciences, Federal University of Bahia School of Medicine, Salvador, Bahia, Brazil; 8 Gonçalo Moniz Research Center, Oswaldo Cruz Foundation, Salvador, Bahia, Brazil; 9 National Institute of Science and Technology in Tropical Diseases (NIST-TD), Salvador, BA, Brazil; 10 Department of Pharmacology, University of Minnesota, Minneapolis, Minnesota, United States of America; 11 Parasitic Biology Graduate Program, Federal University of Sergipe, São Cristóvão, Sergipe, Brazil; U.S. Food and Drug Administration and Center for Biologics Evaluation and Research, UNITED STATES OF AMERICA

## Abstract

**Background:**

Cutaneous Leishmaniasis (CL) is a neglected tropical disease endemic in Brazil. Morbidity and disabilities caused by CL lesions require an analysis of a Global Burden of Disease (GBD), which would help discern the impact on the Brazilian population. Herein, we assess the burden of CL and its spatial and temporal patterns in Brazil between 2001 and 2021.

**Methodology/Principal findings:**

We estimated rates per 100,000 population for years lived with disabilities (YLD), years of life lost prematurely (YLL) and disability-adjusted life years (DALY) for each year of the study, sex assigned at birth, age group, and for each municipality in Brazil. In addition, the relative changes in these metrics over time for each region and sex were determined, as well as temporal trends using segmented joinpoint regression models. Using spatiotemporal analysis tools, we created choropleth maps representing DALY, YLD and YLL for three distinct periods (P1 = 2001 to 2007; P2 = 2008 to 2014; P3 = 2015 to 2021). These maps were constructed to visualize the inferences from Bayesian spatial statistics and Moran’s autocorrelation using the Poisson model. The data were obtained from the DATASUS database. Although the global burden of CL has reduced over two decades, with the continual high impact among adults aged 20 to 39 years. In turn, YLL increased over time in 40-year-old populations, while among the elderly (>60 years old) this rate almost doubled from 2010 to 2021. Regarding the region of residence, we observed an average increase of 28% in YLL in Southeast, South and Central-West. Furthermore, the global burden of CL does not have a random spatial distribution, since there was a high-risk clustering of YLD in the north of the country. Interestingly, the YLL showed a vast geographic expansion through Brazilian territory.

**Conclusions:**

This study provides a comprehensive analysis of the burden of CL in Brazil, pointing out areas of highest disease burden, where control and surveillance efforts should be undertaken.

## Author summary

Previous studies on the burden of Cutaneous Leishmaniasis (CL) have focused on short-term investigations and concomitant with visceral leishmaniasis (VL). This might limit a more comprehensive understanding of CL dynamics. Furthermore, in Brazilian research, there is a lack of linkage between studies and the Global Burden of Disease (GBD) along with spatial and temporal analysis techniques, especially in CL. Our data showed a reduction in DALY and YLD rates. However, there was a significant increase in YLL, predominantly among adults and the elderly, and in previously unrecorded regions. It is important to emphasize the geographical disproportion of the YLD rate, given the formation of high-risk clusters in the North and Central-West regions. These results have significant implications for public health, as well as providing valuable information for directing efforts and resources. Studying the burden of CL in Brazil is crucial because it is among the countries with the highest number of global cases. Therefore, linking this study to spatial analysis has provided new insights and territorial patterns, as well as previously unknown manifestations. Additionally, it serves as a global benchmark that can help not only Brazil but also other endemic countries understand the dynamics of this disease and determine which political and surveillance measures can be taken.

## Introduction

Cutaneous leishmaniasis (CL) is chronic and neglected tropical disease (NTD) caused by intracellular protozoa of the genus *Leishmania* [1]. Clinically, CL can be grouped into localized (LCL) or diffuse (LCD) cutaneous leishmaniasis and mucocutaneous leishmaniasis (MCL), depending on the species of the parasite and the host’s immune response [2]. These conditions are marked by skin lesions, which generate social stigma and reinforces poverty in the affected population and psychological distress [3].

According to the World Health Organization, it is estimated that more than 1 million new cases of CL occur annually in endemic areas [4] Although CL has a worldwide distribution, 85% of registered cases are concentrated in the Americas, with Brazil having the highest number of cases in the world, followed by Colombia and Peru [5]. Additionally, CL is considered one of the most significant public health concerns in the country, with high incidences affecting all age groups and has different transmission mechanisms [6,7].

In Brazil, therapies targeting CL have failure rates of 49.2% to 73.7%, which is contingent on drug dose, parasite strain, and geographic region. In addition to treatment, CL faces other challenges such as the implementation of vector control strategies, diversity of parasite reservoirs, and environmental changes [8,9]. Although therapeutic interventions are effective, access to treatment remains low in coverage and adherence due to the toxicity medications and serious adverse effects [10,11].

Importantly, the Global Burden of Disease (GBD) is a worldwide epidemiological tool that comprises mortality and morbidity from disabilities, illnesses and injuries. The goal of GBD is to measure and analyze population health loss at comparable levels with the aim of eliminating disparities and improving health systems [12,13]. This highly relevant tool is instrumental for assessing the impact of diseases in the context of spatial and temporal analyses. This made it possible to identify the critical regions for the CL load in Brazil and its evolution over the 21 years analyzed.

Notably, a territorial expansion of the impact of the disease was observed, especially in terms of years of life lost (YLL) prematurely, highlighting the significant mortality caused by CL. These findings are crucial to guide CL surveillance and for implementation of control actions to mitigate disease burden. The need to utilize less aggressive therapeutic strategies is also highlighted, especially for elderly populations.

## Methods

### Study area

The Federative Republic of Brazil has approximately 204 million inhabitants (2022 census) and a territory area of 8,510,417,771 km^2^, with population density of 23.86 inhabitants/km^2^ [14]. Considering the territorial extension, Brazil is the largest country in Latin American and the fifth largest in the world. Also, the countryis politically and administratively divided into 27 federal units (26 states and the Federal District) and 5,570 municipalities. The federal units are grouped into five geographic regions: Central-west, Northeast, North, Southeast, and South [14].

### GBD overview

The Global Burden of Disease Study (GBD) is a systematic scientific assessment that assess the impact of a disease and compare the health of the population through indicators composed by morbidity and mortality measures: Years Lived with Disability (YLD), Years of Life Lost (YLL) and Disability-Adjusted Life Years (DALY). The GBD is a project developed by The Institute for Health Metrics and Evaluation (IHME) and the general methodological approaches used by GBD 2019 to estimate the metrics are detailed in previous [12,13].

The estimates of morbidity and mortality are provided annually by GBD 2019 for 369 diseases and injuries, 87 risk factors for 204 countries and territories from 1990 [12] to 2019Leishmaniasis are within the level 2 category neglected tropical disease (NTDs) and malaria, consisting of 20 prioritized infectious and parasitic diseases prioritized by WHO. Among parasitic diseases, leishmaniasis is divided into CL and VL [15].

### Data sources and case definition

This work consists of an observational study of an ecological nature, which uses a times series (2001 to 2021), which describes the metrics of GBD for CL and MCL, in Brazil according to time, sex assigned at birth and age. Data regarding mortality as well as the generation of YLL estimates in Brazil are available in the Brazilian Mortality Information System (Sistema de Informação sobre Mortalidade—SIM). Compliant with GBD, each death is attributed to a single underlying cause; the cause that initiated the series of events leading to death, in accordance with the international classification of Diseases (ICD) principles. The morbidity data used to carry out the YLD estimates correspond to the Information and Diseases and Notification System (Sistema de Informação de agravos e Notificação–SINAN) and to the DALY both databases were used. In this study, the prevalent and fatal cases of CL and CML were used, and we decided to unite the CL and CML bank into a single data, since both have the same disease burden. Unspecified Leishmaniasis and VL cases were not included.

In the GBD, data corrections are performed for underreporting of mortality and redistribution of garbage codes for defined causes based on the GBD redistribution algorithms. Garbage codes are assigning causes of death [16]. However, for CL, GBD assumed that mortality is null over the years, thus having the same values for YLD and DALY [17]. Though, in this work, the authors found it relevant to calculate the YLL for Brazilian municipalities.

In summary, the YLL is the product of the number of deaths and standard life expectancy, this calculation is performed for each age group and sex. The YLD is the product of the prevalence of CL cases by the weight attributed to this condition. The disability weight reflects the severity of the health loss associated with the disease and is presented on a scale which varies from 0 (perfect health) to 1 (equivalent to death)[18]. The DALY is the sum of YLD and YLL. The three metrics were adjusted for a rate of 100,000 inhabitants.

### Metrics presentation

After calculating the rate per 100,000 inhabitants for the three metrics (YLD, YLL and DALY) in the female and male population, for each year of the study and in the different regions of the country, the percentages of relative changes were calculated for three-time cuts, period 1 (P1) (2001–2007), period 2 (P2) (2008–2014) and period 3 (P3) (2015–2021). The metrics were presented with their respective 95% confidence intervals (95% CI). Excel 17.0 (2019) was used for the organization and storage of data, and for preparation the figures, percentages of relative change, was used the Graph Pad Prism 8.0.

### Temporal trend analysis

Temporal trends of YLD, YLL and DALY rates by sex assigned at birth, age group, and Brazilian regions, were assessed by the segmented linear regression method, using the Joinpoint Regression Program (version 4.9.0.0). This method has been used in previous studies [19,20] and allows verifying changes in the trend of the variables over time, Thereby, time series can show an increasing, decreasing, or stable trend [21].

We applied the Monte Carlo permutation test to select the best segment from each model using 9999 permutations. We also calculated the annual percentage change (APC) for each period. The temporal trends were statistically significant, showing in APC the p-value <0.05 and its 95% CI. The results were interpreted as follows: time trends with significant and positive APC were considered increasing; significant and negative APC, decreasing; when there was no significant result, the trends were classified as stable [21].

### Spatial analysis

The maps of Brazil were constructed representing the crude YLD, YLL, and DALY rates of prevalence and fatal cases for CL according to the three periods mentioned above (P1, P2 and P3). Thereafter, we smoothed the rates through the Empirical Local Bayesian method to correct random fluctuations and provide greater stability to the obtained rates. Method already described in other works [19,20].

Subsequently, we also calculated the Global Moran Index (GMI) to check for spatial autocorrelation. This analysis identifies correlation of a variable with itself, varying between -1 and +1. Then, we evaluated the occurrence of local spatial autocorrelation, using the Local Moran Index (Local Indicators of Spatial Association–LISA), whose objective is to verify the existence of municipalities that present similar patterns through clusters of high and low risk and of transition, generating four quadrants: Q1 (high/high) and Q2 (low/low), indicate municipalities with similar values among their neighbors and Q3 (high/low) and Q4 (low/high) indicate municipalities with different values between neighbors and no spatial association [22]. Results with a significance level of p-value <0.05 were considered significant. For these analyses, we used the TerraView software, version 4.2.2, and the maps were constructed using the QGIS software, version 3.18.

The Population estimates and the digital cartographic grid (in shapefile extension), segmented by municipalities and states, of the Universal Transversal Mercator (UTM) system, horizontal Terra Datum model (SIRGAS 2000), were collected from the databases of the Brazilian Institute of Geography and Statistics (IBGE) [23].

## Results

**Fig 1:**
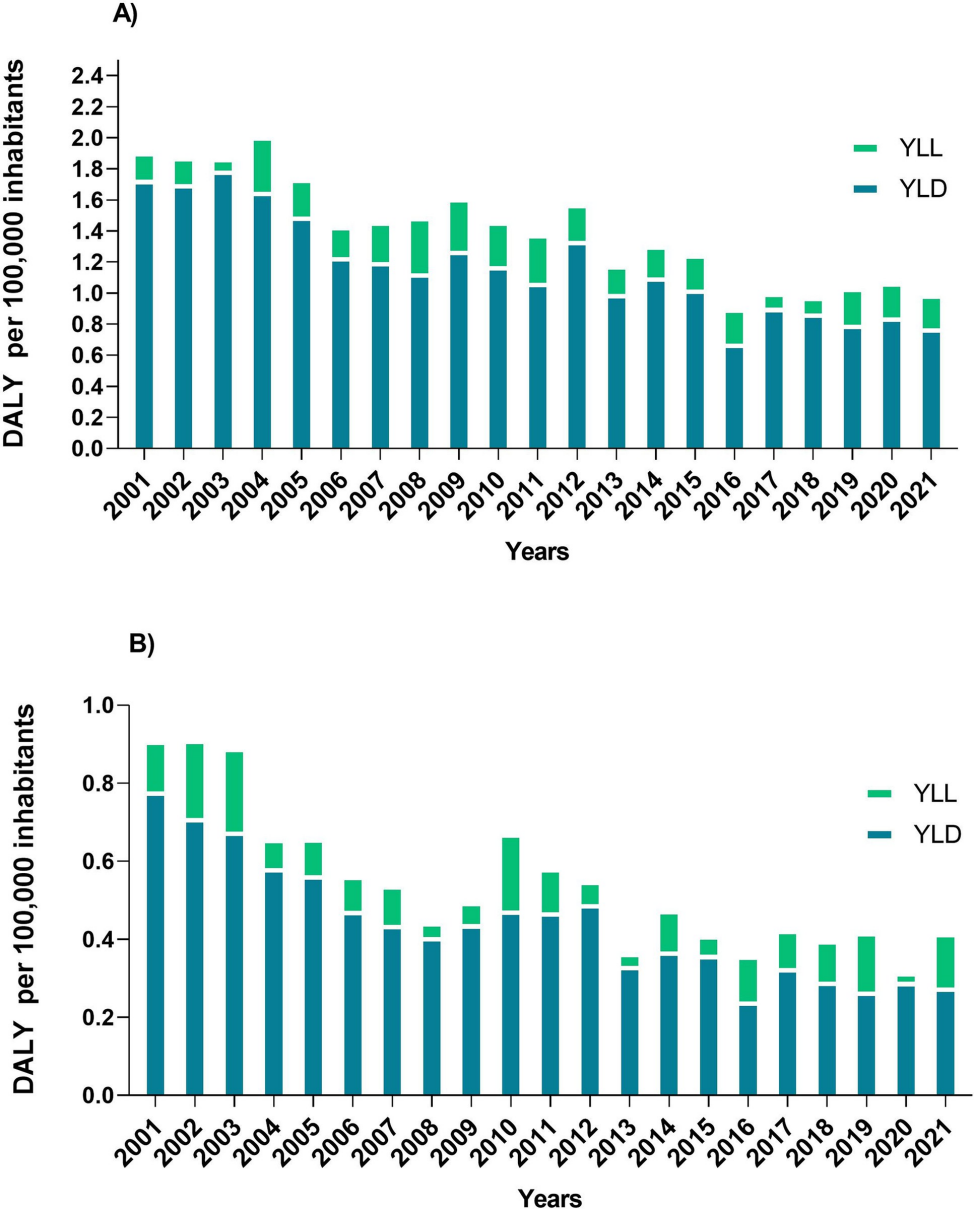
Annual rates per 100,000 inhabitants of disability-adjusted life years (DALY) along with the contribution of years of life lost to premature death (YLLs) and years lived with disability (YLDs) among men (A) and women (B). Brazil, 2001–2021.

The main metrics on CL load and its variations between 2001 and 2021 are detailed in Fig 1. There was a reduction in the DALY rate in both sex over the years, as well as the contribution of YLD. However, it is evident that among males, the rates are higher when compared to the female population. In 2001, the YLD for men reached 1.7/100,000 inhabitants and the YLL 0.17/100,000 inhabitants. For women these metrics were 0.77 and 0.13/100,000 inhabitants, respectively. In 2021, YLD and YLL rate among men dropped to 0.77 and 0.21/100,000 inhabitants. Similar patterns were also found in women, where the YLD was 0.21 and the YLL 0.14/100,000 inhabitants (Fig 1).

**Fig 2:**
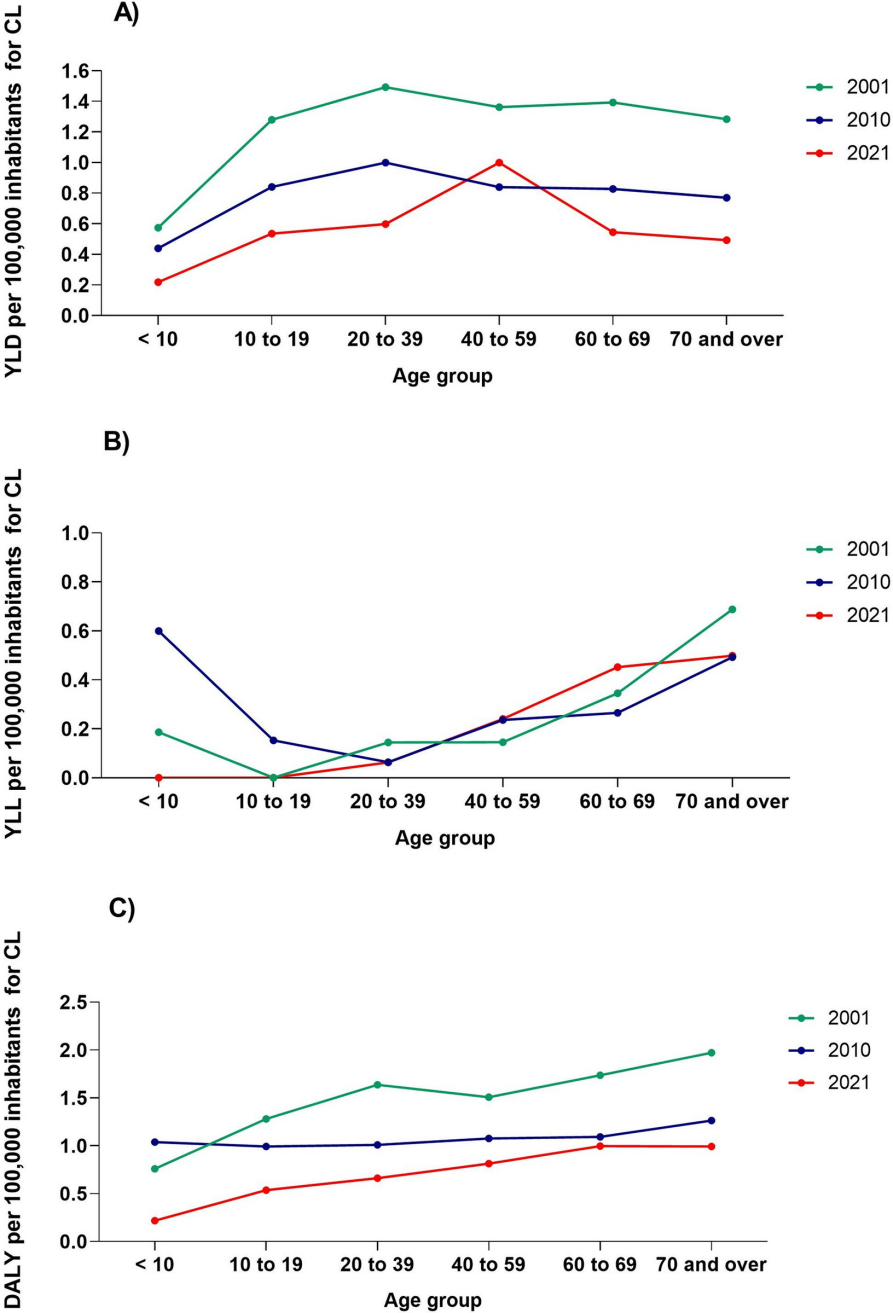
Rates per 100,000 inhabitants for Cutaneous Leishmaniasis (CL) with disability by age group, Brazil in 2001, 2010 and 2021. (A) Years lived with disability (YLD). (B) Years of life lost (YLL). (C) Disability-adjusted life years (DALY).

An increase in YLD rates was spotted largely in individuals until 39 years of age, with rates found of 1.49, 1.0 and 0.59/100,000 inhabitants in 2001, 2010 and 2021, respectively (Fig 2A). In 2021 there was a total reduction of YLL in < 10 years. However, in age groups from 40 years old this metrics increased gradually. In elderly people of 60 to 69, rates went from 0.26/100,000 inhabitants in 2010, to 0.45/100,000 inhabitants in 2021 (Fig 2B). Regarding the DALY rate, in general, a reduction was observed in all age groups, with the adult population aged 20 to 69 being the most affected (Fig 2C).

When analyzing the temporal trends during the study period (2001 to 2021), we observed an increasing trend in YLD for men (APC: 32.5; CI95%: 20.4 to 45.9) and women (APC: 52.9; CI95%: 36.6 to 71.1). Meanwhile, we observed decreasing trends on YLD rates for all Brazilian regions. The YLL remained stable in most regions, but the Northeast showed a decreasing trend (APC: - 5.0; 95% CI: -8.3 to -1.6) (Table 1).

**Table 1:** Time trends of rates per 100,000 inhabitants of years of life lost (YLL), years lived with disability (YLD) and disability-adjusted life years (DALY) by sex and regions, Brazil, 2001–2021.

Metrics	Period	Segmented period APC (CI95%)	Trend	p-value
**Sex**				
**Males**				
YLD	2001–2021	32.5* (20.4 to 45.9)	Increasing	<0.001
YLL	2001–2021	-1.2 (-3.8 to 1.4)	Stable	0.343
DALY	2001–2021	-3.6* (-4.3 to -2.9)	Decreasing	<0.001
**Females**				
YLD	2001–2021	52.9* (36.6 to 71.1)	Increasing	<0.001
YLL	2001–2021	-1.7 (-5.0 to 1.7)	Stable	0.304
DALY	2001–2021	-4.4* (-5.5 to -3.3)	Decreasing	<0.001
**Region of Residence**				
**YLD**				
North	2001–2021	-4.0* (-5.2 to -2.8)	Decreasing	<0.001
Northeast	2001–2021	-6.2* (-7.7 to -4.6)	Decreasing	<0.001
Southeast	2001–2021	-3.6* (-4.1 to -1.1)	Decreasing	<0.002
South	2001–2021	-7.1* (-8.6 to -5.5)	Decreasing	<0.001
Central-West	2001–2021	-5.6* (-6.6 to -4.7)	Decreasing	<0.001
Brazil	2001–2021	-4.4* (-5.2 to -3.7)	Decreasing	<0.001
**YLL**				
North	2001–2021	-2.0 (-5.3 to 1.4)	Stable	0.223
Northeast	2001–2021	-4.5*(-7.4 to -1.5)	Decreasing	0.006
Southeast	2001–2021	-1.4 (-6.6 to 4.0)	Stable	0.586
South	2001–2021	3.0 (-2.1 to 8.5)	Stable	0.235
Central-West	2001–2021	-2.1 (-6.2 to 2.1)	Stable	0.307
Brazil	2001–2021	-1.1 (-3.1 to 0.3)	Stable	0.108
**DALY**				
North	2001–2021	-3.7* (-4.8 to -2.6)	Decreasing	<0.001
Northeast	2001–2021	-5.8* (-7.4 to -4.2)	Decreasing	<0.001
Southeast	2001–2021	-1.9* (-3.7 to -0.1)	Decreasing	0.040
South	2001–2021	-3,4* (-6.2 to -0.6)	Decreasing	0.020
Central-West	2001–2021	-4,6* (-5.8 to -3.4)	Decreasing	<0.001
Brazil	2001–2021	-3.9* (-4.6 to -3.3)	Decreasing	<0.001

APC–Annual Percent Change; CI–Confidence Interval.

*p-value <0.05.

Considering age groups, all demonstrated a decreasing temporal trend of YLD and DALY. Differently, for YLL, we observed a stable trend in all groups (S1 Appendix).

In general, the metrics of the three periods analyzed by relative change reduced in men and women. The YLD of men from P1 to P2 increased by 29.29%. However, between P1 and P3 there was a reduction of 10.34%. A decrease in YLD and DALY was observed in the percentage variation in the regions of residence. However, it is important to emphasize that the YLL in the Southeast region in the three-time cuts showed an increase, and from P2 to P3 the increase was 20.56%. In the South region had an increase of more than 100% from P2 to P3 and 40.71% from P1 to P3. The Central-West region also suffered from high YLL, demonstrating P1 to P3 the increase reached 22.68% (Table 2).

**Table 2:** Rates per 100,000 inhabitants and relative change of years lived with disability (YLD), in years of life lost (YLL) and disability-adjusted life years (DALY) by sex and regions of Brazil in the periods 2001–2007 (P1), 2008–2014 (P2), 2015–2021 (P3).

Metrics	Rate per 100,000 (95%CI)			Relative change (%)		
	P1	P2	P3	P1 x P2	P2 x P3	P1 x P3
**Sex**						
**Males**						
YLD	1.54	1.15	0.84	-25.22	-27.11	-45.49
	(1.3–1.7)	(1.0–1.2)	(0.7–0.9)			
YLL	0.21	0.27	0.19	29.29	-30.65	-10.34
	(0.1–0.2)	(0.2–0.3)	(0.1–0.2)			
DALY	1.75	1.42	1.03	-18.62	-27.78	-41.23
	(1.5–1.9)	(1.2–1.5)	(0.9–1.1)			
**Females**						
YLD	0.60	0.42	0.29	-29.51	-31.10	-51.44
	(0.4–0.7)	(0.3–0.4)	(0.2–0.3)			
YLL	0.12	0.08	0.09	-32.95	-13.29	-24.00
	-	-	-			
DALY	0.73	0.51	0.39	-30.11	-23.57	-46.59
	(0.5–0.8)	(0.4–0.6)	(0.3–0.4)			
**Region of Residence**						
**YLD**						
North region	0.82	0.45	0.35	-45.14	-21.64	-57.01
	(0.6–1.0)	(0.3–0.5)	(0.2–0.4)			
Northeast Region	0.49	0.28	0.15	-42.96	-45	-68.62
	(0.3–0.6)	(0.2–0.3)	(0.1–0.1)			
Southeast region	0.15	0.11	0.12	-26.55	-15.62	-15.08
	-	-	-			
South region	0.16	0.09	0.06	-42.00	-35.16	-62.39
	(0.1–0.2)	(0.0–0.1)	-			
Central-West Region	0.12	0.06	0.05	-44.97	-25.65	-59.09
	(0.0–0.1)	-	-			
Brazil	1.07	0.78	0.56	-26.74	-28.58	-47.68
	(0.9–1.2)	(07–0.8)	(0.4–06)			
**YLL**						
North region	0.42	0.62	0.37	45.88	-39.02	-11.04
	(0.2–0.6)	(0.3–0.8)	(0.2–0.4)			
Northeast Region	0.29	0.25	0.1	-13.69	-48.15	-55.25
	(0.1–0.3)	(0.1–0.3)	(0.0–0.1)			
Southeast region	0.07	0.08	0.08	19.01	1.29	20.56
	(0.0–0.1)	(0.0–0.1)	(0.0–0.1)			
South region	0.04	0.02	0.06	-40.89	138.10	40.71
	-	-	(-0.0–0.1)			
Central-West Region	0.29	0.32	0.35	10.98	10.54	22.68
	(-0.0–05)	(0.0–0.5)	(0.1–0.5)			
Brazil	0.17	0.18	0.14	7.08	-23.25	-17.809
	-	(0.1–0.2)	-			
**DALY**						
North region	5.81	4.56	3.30	-21.53	-27.53	-43.14
	(4.8–6.7)	(4.0–5.0)	(2.9–3.6)			
Northeast Region	1.50	1.16	0.63	-22.56	-45.80	-58.02
	(1.0–1.9)	(0.9–1.4)	(0.5–0.7)			
Southeast region	0.32	0.25	0.26	-22.56	2.45	-20.18
	(0.2–0.4)	(0.1–0.3)	(0.2–0.3)			
South region	0.22	0.12	0.13	-43.02	2.40	-41.65
	(0.1–0.2)	(0.0–0.1)	(0.0–0.1)			
Central-West Region	2.77	1.92	1.46	-30.71	-23.69	-47.13
	(2.1–3.3)	(1.5–2.3)	(1.2–1.6)			
Brazil	1.24	0.97	0.70	-22	-27.56	-43.50
	(1.0–1.4)	(0.8–1.0)	(0.6–0.7)			

CI–Confidence Interval

**Fig 3:**
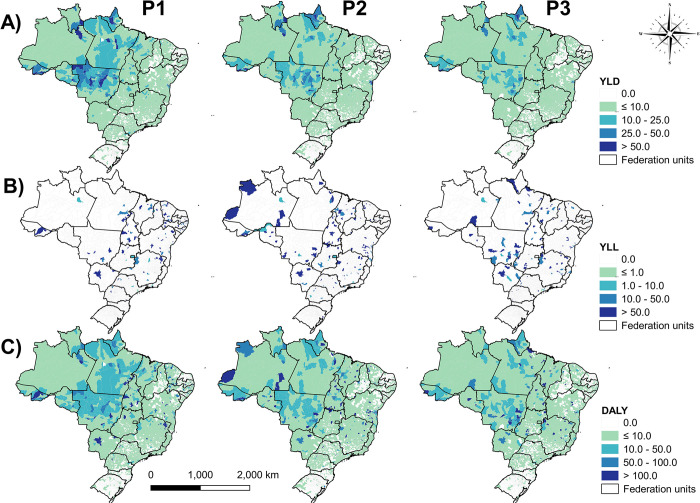
Spatial and space-time analysis of the gross rate distribution of years lived with disability (YLD) (A), years of life lost (YLL) (B) and disability-adjusted life years (DALY) (C) in Brazil in the periods 2001–2007 (P1), 2008–2014 (P2), 2015–2021 (P3).

Results revealed a wide distribution of YLD and DALY in the three periods, with clusters of high rates, mainly in the North and Central-West regions of Brazil, which reduced as the years progressed (Fig 3A and 3C). The crude YLL rate presented smaller and punctual distributions with clusters of high rates in some municipalities (Fig 3B).

**Fig 4:**
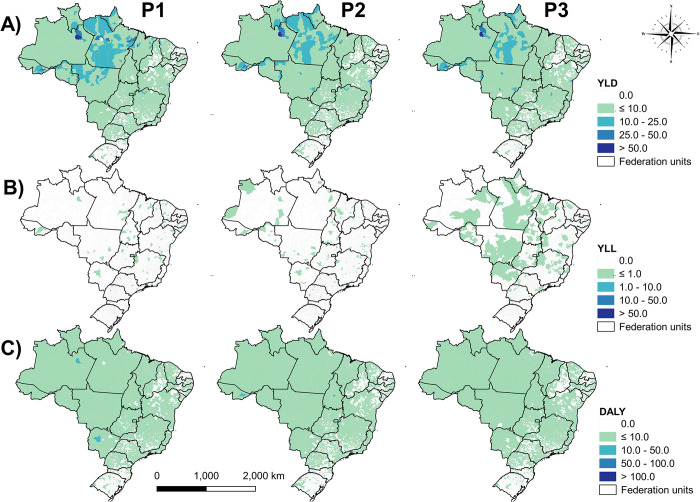
Spatial and spatiotemporal analysis of the distribution of smoothed rates of years lived with disability (YLD) (A), years of life lost (YLL) (B) and disability-adjusted life years (DALY) (C) in Brazil over the periods 2001–2007 (P1), 2008–2014 (P2), 2015–2021 (P3).

When analyzing the smoothed rates of the metrics, we observed that YLD remained with a wide geographic distribution, as did DALY, and also with the presence of clusters of high rates that continued to decrease over time P1, P2 and P3 (Fig 4A and 4C). On the other hand, the distribution of low YLL rates has been widened across the country (Fig 4B).

**Fig 5:**
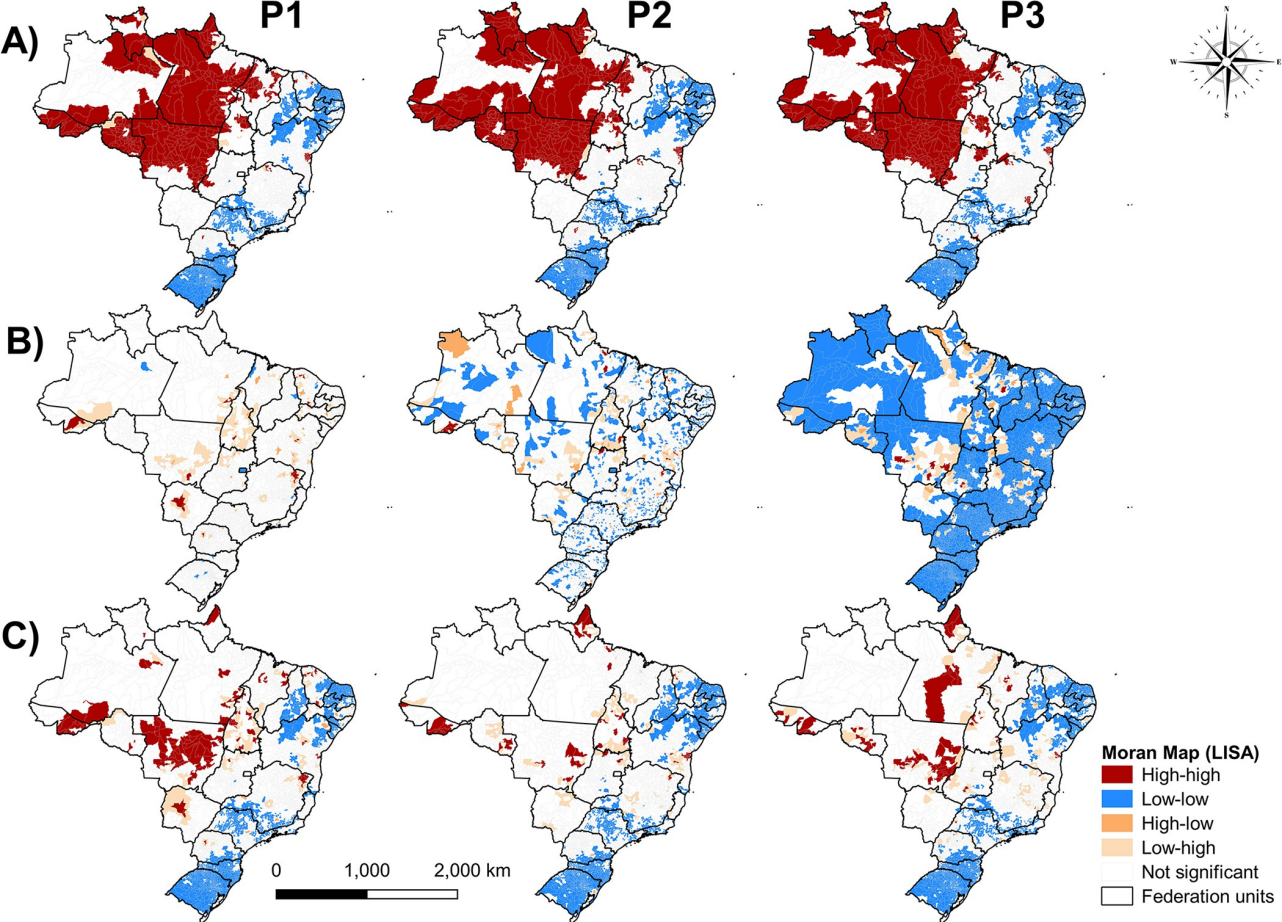
Spatial autocorrelation using the Univariate LISA analysis; (LISA) of years lived with disability (YLD) (A), years of life lost (YLL) (B) and disability-adjusted life years (DALY) (C) in Brazil over the periods 2001–2007 (P1), 2008–2014 (P2), 2015–2021 (P3).

In the spatial autocorrelation analysis using the univariate GMI, we revealed the existence of spatial dependence of YLD in municipalities with similar patterns among all periods (P1 = 0.532, *p*-value = 0.001; P2 = 0.554, *p*-value = 0.001; P3 = 0.564, *p*-value = 0.001) (Fig 5A). In all periods, a high-risk cluster was observed that was concentrated in the North region, reaching practically all states and the state of Mato Grosso (MT) in the Central-West region. In the Northeast and South regions, low-risk clusters predominated; in the Southeast, the states that presented the most low-risk clusters were São Paulo (SP) and Minas Gerais (MG) (Fig 5A).

Regarding YLL, the occurrence of spatial dependence and existing autocorrelation was low or non-existent (P1 = 0.015, p-value = 0.56; P2 = 0.008, p-value = 0.083; P3 = -0.002, p-value = 0.5) (Fig 3B). However, the predominance of a low/high transition zone with few high-risk clusters was demonstrated in P1. Differently, in P2 there were still transition zones, but with territorial spread of small low-risk clusters, which were intensified in P3 throughout Brazil. Also, in P3 smaller high-risk clusters are observed, mainly in MT and Maranhao (MA) (Fig 5B).

Although the spatial patterns found for DALY remained similar between periods, with the presence of high-risk clusters in the North and low risk for the rest of the country, there was the formation of high-risk clusters located in P1 (GMI = 0.039, p -value = 0.04) that disappeared in P2 (GMI = 0.021, p-value = 0.02) in some northern states. However, in P3 (GMI = 0.009, p-value = 0.112) there was a reappearance in Manaus (AM) and MT, in addition to transition clusters in some other states (Fig 5C).

## Discussion

To the best of our knowledge, this is the first study to demonstrate a current and detailed analysis of the spatial and temporal patterns of the burden of CL in Brazil. Our data highlight that the geographic inequalities and the demographic profile currently associated with CL disease, as well as the progress that Brazil has achieved over the last two decades. Remarkable, the main findings were changes in the burden of the disease and the decrease in YLD and DALY rates over time, while the YLL rate showed important fluctuations, mainly affecting individuals over 40 years of age. Additionally, the highest rates observed, as well as high-risk clusters, are predominantly concentrated in the North and Central-West regions of the country.

Generally, in Brazil, adult men have been more affected by CL, forming a consistent epidemiological pattern, although there has been a reduction in numbers, they remain the most affected group due to occupational and behavioral factors that place them in more frequent contact with disease vectors [7]. As a result, the burden of the disease in this group is higher compared to women. Although CL has the potential for spontaneous healing, the infection can result in disabilities and disfiguring scars, in addition to manifesting various forms and complications that are challenging to treat [24].

In Brazil, the recommended treatment for CL, regardless of the species, continues to be meglumine antimoniate. However, for individuals over 50 years of age with specific clinical conditions, the recommended drug is liposomal amphotericin B [25]. Although it presents lower toxicity, some patients have suffered adverse effects such as kidney damage [26]. Furthermore, its use faces limitations due to its high cost [26,27]. Another important factor that has been highlighted in the population over 50 years of age is the occurrence of CML, as indicated by Monachese and colleagues [28] in a national study, CML affected 16.8% of patients over 75 years of age; in a separate survey carried out in Bolivia, 36% of patients over 65 years of age developed CML [29]. The recurrence of the mucocutaneous form in these individuals suggests that immunosenescence [30] added to the difficulties of treatment, it can influence clinical complications and lethality. Taken these data together, the authors recommend that the significant increase in YLL in this age group in recent years in Brazil is possibly related to the evidence highlighted above.

When assessing time trends by age, we identified that the YLL rate remained stable in most age groups, with a decreasing trend only for children under ten years old, unlike YLD and DALY which had a decreasing behavior in all age groups and in regions of residence, the downward trend in these metrics may be associated with improvements in control and surveillance actions or even individual protection measures against the vector [7].

Although the percentage variation in the YLD and DALY metrics, a decreasing trend was observed over the years. Nevertheless, what stands out once again is the increase in the YLL rate in the Southeast, South and Central-West regions. Although GBD assumes zero mortality for CL [16], in Brazil, 443 deaths were reported, from 2001 to 2021, (Data not mentioned) therefore, it is clear that mortality due to CL deserves to be discussed and prioritized, above all, as demonstrated in this study in the elderly population, which has been losing years of life prematurely. CL is the most common form in Brazil, however, the CML form was slightly more reported in the South, Southeast and Center-West [7], thus suggesting that the more severe form of the disease [15] has contributed significantly to the reduction in years of life in this population.

CL is a disease with wide distribution in Brazil, due to the diversity of vectors and etiological agents that, linked to environmental conditions, end up favoring the onset of the disease mainly in the Legal Amazon region, which encompasses the seven states of the North region, plus Mato Grosso (Midwest) and Maranhao (Northeast) [31]. In fact, as pointed out in this work, after smoothing, the highest rates of disease burden are concentrated in the North region, however, the occurrence of the disease occurs throughout the country throughout the analyzed period, requiring constant surveillance and improvement of the notification system in all five regions, including in non-endemic areas such as the Northeast.

In endemic areas, the occurrence of CL is directly influenced by a combination of climatic, environmental, cultural, and socioeconomic factors [31], that in turn contribute to the constant burden of leishmaniasis in the North and Central-West. The existence of spatial dependence of YLD in these regions may have been possibly caused by these conditions. High-risk clusters for YLD were consistently identified in the area mentioned above during the three periods analysed, this region is mainly marked by the presence of the species *L*. *guyanensis* [32] responsible for causing single or disseminated lesions in the patient, the latter being the most frequent resulting from several sandfly bites or lymphatic metastasis [33] and although the lesions have a high parasite load, which facilitates diagnosis, patients with this clinical form tend to have a lower response to treatment with antimonials [34].

The North Region, which is home to the largest concentration of descendants of original peoples, has faced challenges related to environmental changes caused by activities such as illegal mining, invasions of indigenous lands by land grabbers and destruction of ecosystems due to the advancement of agribusiness. These actions can lead to ecological imbalances, increasing contact between sandflies and their hosts. Furthermore, in the Amazon region access to diagnosis and treatment is limited, largely due to the isolation of communities and the precarious financial condition of many patients also constitutes a barrier, preventing them from travelling to larger centres in search of better services of health [34]. Obviously, these impasses interfere with the patient’s healing and mean that these individuals are living with the disease for a long time, influencing their personal and professional routine, in addition to self-esteem and suffering with the gradual loss of health.

DALY shows similar behaviour to YLD, because the latter is the metric that most contributes to inferences about the disease burden of CL. On the other hand, YLL showed a wide distribution but there was no spatial dependence, which means that there is still no certainly a characteristic factor of a certain region contributing to the loss of years of life prematurely, Brazil as a whole presents a low-risk cluster for YLL, but the possibility of these areas evolving to high risk should not be ruled out, since, due to due to the migratory flow leaving the Amazon region for the Central-West and South, MCL took on large geographic proportions, with notification records throughout the country [35,36].

This study has some limitations. First, the use of secondary data that depends on the coverage of information systems and the quality of records in different Brazilian municipalities is a major challenge for ecological studies. Second, to measure the burden of the disease, data from two banks is necessary, which could therefore have its value underestimated or overestimated due to the lack of interoperability of the systems. Lastly, some regions such as the North and Northeast still need improvements in relation to case registration coverage, however, health databases have made advances in recent years and SINAN covers both public and private services, making it more complete. In addition, the diagnosis and treatment of leishmaniasis are available free of charge in the Unified Health System (UHS).

Research that employs geoprocessing and spatial analysis techniques allows the understanding of the problem under study in addition to demonstrating its behavior in space and time. As pointed out in this work with the burden of CL, in which it was possible to identify for the first time which regions of Brazil are being most affected in terms of health loss and the emergence of new clusters. These results serve as support for decision-making in critical areas, as well as validating previously used strategies that helped reduce CL transmission in certain areas.

It is important to highlight that the cutaneous and mucocutaneous manifestations of leishmaniasis have different clinical approaches. Patients with cutaneous leishmaniasis generally require smaller doses of medication, have a shorter treatment period, and most often respond well to treatment. On the other hand, those with the mucocutaneous form require longer treatment, have a less efficient immune response and face a more aggressive form of the disease, which can be relapsed [37]. In this study we worked with the unification of cases of CL and CLM as both present the same weight, however, we suggest for the next GBD estimates to consider different weights for the clinical forms and separate analyses, as well as the measurement of YLL, given the mortality recorded in Brazil.

The burden assessment was extremely important to identify the population vulnerable to the disease and understand its impact on the health of Brazilians. From our analyses, it is possible to clearly state that mortality due to CL is an expanding problem in Brazil and needs to be prioritized, especially in the elderly population, although the burden of CL has decreased over the years, the same areas remain affected. There is an urgent need for more investment in the discovery of new drugs and treatments, vaccines and early diagnosis. Furthermore, the results reinforce the importance of maintaining a transparent and high-quality reporting system.

## Declaration of generative AI and AI-assisted technologies in the writing process

During the preparation of this work the authors used the GPT-3 model, developed by OpenAI in order to exclusively to improve the quality of language English text with caution. After using this tool/service, the authors reviewed and edited the content as needed and takes full responsibility for the content of the publication.

## Supporting information

S1 Appendix Time trends of rates per 100,000 inhabitants of years lived with disability (YLD), years of life lost (YLL) and disability-adjusted life years (DALY) by age group, Brazil, 2001–2021. (DOCX)
