# Supplementary material for: Spatial and temporal modeling of the global burden of Cutaneous Leishmaniasis in Brazil: A 21-year ecological study
Source: PLoS Negl Trop Dis. 2024 Nov 20;18(11):e0012668. doi: 10.1371/journal.pntd.0012668 (PMC11578532; doi:10.1371/journal.pntd.0012668)
Supplement: S1 Appendix — (DOCX) [file pntd.0012668.s001.docx]

**S1 Appendix**. Time trends of rates per 100,000 inhabitants of years lived with disability (YLD), years of life lost (YLL) and disability-adjusted life years (DALY) by age group, Brazil, 2001-2021.

| **Metrics** | **Period** | **Segmented period APC (CI95%)** | **Trend** | **p-value** |
| --- | --- | --- | --- | --- |
| **YLD** |  |  |  |  |
| < 10 years | 2001 - 2021 | -3.5* (-5.4 to -1.6) | Decreasing | 0.001 |
| 10 - 19 years | 2001 - 2021 | -4.6* (-5.5 to -3.6) | Decreasing | <0.001 |
| 20 - 39 years | 2001 - 2021 | -4.6* (-5.4 to 3.9) | Decreasing | 0.001 |
| 40 - 59 years | 2001 - 2021 | -4.4*(5.1 to -3.6) | Decreasing | <0.001 |
| 60 - 69 years | 2001 - 2021 | -5.2* (-6.0 to -4.3) | Decreasing | <0.001 |
| 70 and over | 2001 - 2021 | -4.6* (-6.0 to -4.3) | Decreasing | <0.001 |
| **YLL** |  |  |  |  |
| < 10 years | 2001 - 2021 | -8.2* (0.7 to 16.2) | Decreasing | 0.033 |
| 10 - 19 years | 2001 - 2021 | -14.5 (-46.2 to 35.8) | Stable | 0.487 |
| 20 - 39 years | 2001 - 2021 | -1.9 (-5.3 to 1.6) | Stable | 0.261 |
| 40 - 59 years | 2001 - 2021 | -0.9 (-4.7 to 3.2) | Stable | 0.637 |
| 60 - 69 years | 2001 - 2021 | -1.2 (-5.2 to 3.0) | Stable | 0.566 |
| 70 and over | 2001 - 2021 | -1.5 (-3.4 to 0.4) | Stable | 0.114 |
| **DALY** |  |  |  |  |
| < 10 years | 2001 - 2021 | -5.3* (-8.0 to -2.6) | Decreasing | 0.001 |
| 10 - 19 years | 2001 - 2021 | -4.5* (-5.5 to -3.6) | Decreasing | <0.001 |
| 20 - 39 years | 2001 - 2021 | -4.6* (-5.3 to -3.9) | Decreasing | <0.001 |
| 40 - 59 years | 2001 - 2021 | -4.7* (-4.7 to -2.4) | Decreasing | <0.001 |
| 60 - 69 years | 2001 - 2021 | -3.4* (-4.8 to -2.0) | Decreasing | <0.001 |
| 70 and over | 2001 - 2021 | -3.0* (-4.0 to -2.1) | Decreasing | <0.001 |

APC – Annual Percent Change; CI – Confidence Interval. *p-value <0.05.
